# Treating Eating: A Dynamical Systems Model of Eating Disorders

**DOI:** 10.3389/fpsyg.2020.01801

**Published:** 2020-07-24

**Authors:** Emily T. Troscianko, Michael Leon

**Affiliations:** ^1^The Oxford Research Centre in the Humanities (TORCH), University of Oxford, Oxford, United Kingdom; ^2^Department of Neurobiology and Behavior, University of California, Irvine, Irvine, CA, United States

**Keywords:** eating disorders, cognitive behavior therapy, behavior, eating speed, dynamical systems, feedback, psychology

## Abstract

Mainstream forms of psychiatric talk therapy and cognitive behavioral therapy (CBT) do not reliably generate lasting recovery for eating disorders. We discuss widespread assumptions regarding the nature of eating disorders as fundamentally psychological disorders and highlight the problems that underlie these notions, as well as related practical problems in the implementation of mainstream treatments. We then offer a theoretical and practical alternative: a dynamical systems model of eating disorders in which behavioral interventions are foregrounded as powerful mediators between psychological and physical states. We go on to present empirical evidence for behavioral modification specifically of eating speed in the treatment of eating disorders, and a hypothesis accounting for the etiology and progression, as well as the effective treatment, of the full spectrum of eating problems. A dynamical systems approach mandates that in any dietary and lifestyle change as profound as recovery from an eating disorder, acknowledgment must be made of the full range of pragmatic (psychological, cultural, social, etc.) factors involved. However, normalizing eating speed may be necessary if not sufficient for the development of a reliable treatment for the full spectrum of eating disorders, in its role as a mediator in the complex feedback loops that connect the biology and the psychology with the behaviors of eating.

## Introduction: What Are Eating Disorders? Mind, Body, or Behavior

### The Cognitive/Behavioral Conflict

Anorexia nervosa (AN), bulimia nervosa (BN), binge eating disorder (BED), and other eating disorders are often considered to be psychological^1^ disorders with physical symptoms. The mind-comes-first perspective is traditionally associated with the psychoanalytic tradition (e.g., Wooldridge, 2018). This perspective is also deeply rooted in the folk psychology of mental illness, which often involves psychologizing explanations of states and behaviors based on a person’s life history (Haslam et al., 2007). Perhaps in part because the psychology-first way of thinking is so instinctive, it is prevalent even in the forms of treatment for eating disorders that reject many psychoanalytical principles – most notably, cognitive behavioral therapy (CBT), one of the most extensively researched and widely practiced treatments for eating disorders.

The great contribution of CBT to the understanding and treatment of “psychological disorders” has been to recognize that such problems are not “just” psychological (with or without prominent physical symptoms): that behavior is always a crucial part of how they begin, persist, and end. As the category name (*eating* disorder) should remind us, this is perhaps even more crucial to acknowledge for eating disorders than for any other “mental health condition.” Ignore or trivialize the repeated everyday actions of binging, purging, delaying one’s eating, weighing one’s food, weighing oneself, checking and rechecking the nutritional information on packets in supermarkets, and all the rest, and you misunderstand the entire disorder. The history of CBT, however, has involved an uneasy oscillation between the behavioral and the cognitive. This pattern has been playing out in CBT for eating disorder treatment since its inception in the 1980s. When CBT was initially used for the treatment of eating disorders, it had a clear behavioral focus that helped people relearn how to eat normally, and this was demonstrably effective (Freeman et al., 1988). However, that emphasis soon gave way to examining primarily the cognitive causes and maintaining factors of eating disorders (Södersten et al., 2017).

The unstable relationship between cognitive and behavioral issues is visible in both the theory and the practice of CBT for eating disorders today. In a widely used CBT treatment manual (Fairburn, 2008), much of the concern is with how to support patients in making rapid and sustainable behavioral change in areas like self-monitoring of eating habits, regular weighing, and cessation of purging. The introduction, however, also describes eating disorders as “essentially ‘cognitive disorders”’ whose “core psychopathology” is the overvaluation of shape and weight and their control: “most features of these disorders appear to be secondary to it and its effects” (2008, p. 12) and “can be understood as stemming directly from it” (p. 18).

This cognitive-over-behavioral hierarchy in core CBT texts – the behaviors must be addressed, but only in service of addressing the cognitions that are the true causes – perhaps helps explain why in the translation of theory and guidelines into clinical practice, a cognitive-*before*-behavioral approach is so widespread. After all, if the primary point is really to change the cognitions, why not just start with them? Glenn Waller (2011) observes that a common assumption in current CBT practice is that if you “start with the cognitions and the emotions,” “behavioral change and physiological recovery will follow.”

The cognitive-before-behavioral practice goes directly against treatment guidelines for eating disorders, which often encourage a behavioral focus designed to effect physical rehabilitation, especially at the start of treatment. The American Psychiatric Association’s *Practice Guideline for the Treatment of Patients with Eating Disorders* (Yager et al., 2006), for instance, includes repeated mention of the benefits of beginning with behavior-focused approaches, especially in treatment of AN: “The goals of nutritional rehabilitation for seriously underweight patients are to restore weight, normalize eating patterns, achieve normal perceptions of hunger and satiety, and to correct biological and psychological sequelae of malnutrition” (Yager et al., 2006, p. 14). The authors clearly state that this behavior-mediated physical rehabilitation should precede psychological work: “Clinical consensus suggests that psychotherapy can be helpful for patients with anorexia nervosa once their malnutrition has been corrected and they have begun gaining weight” (p. 45). The recommendations for BN also emphasize the importance of early behavioral normalization around binge and purge habits. This advice is striking for originating in a psychiatric setting, but it is even more striking that these guidelines are not widely followed. The idea that psychologically focused therapy should precede eating treatment, because the psychological problems are the original cause and/or the primary maintaining factor for the disorder, remains widespread. Squarely behavior-focused approaches are correspondingly rare: “Evidence-based CBT is behavioral at its core…but it is uncommon in everyday practice” (Waller, 2011).

As Waller’s remark implies, there is a problem with the drift from the behavioral to the cognitive: what is currently common in everyday practice is on the whole not working well. Mainstream treatments are often unsuccessful in treating either the disordered eating behaviors or the psychological problems associated with them. Outcome studies and systematic reviews of psychotherapeutic and inpatient treatments (some also including drug treatment) for eating disorders typically show that although some patients may experience symptomatic improvement in the short term, dropouts from treatment are high, many patients relapse after the end of treatment, long-term outcomes are often unknown, and deaths during or after treatment are not uncommon, especially in AN (Steinhausen, 2002, 2009; Lock et al., 2006; Berkman et al., 2007; Keel and Brown, 2010; Kass et al., 2013; Watson and Bulik, 2013; Södersten et al., 2017, 2019a; Zeeck et al., 2018; Fisher et al., 2019).

A recent review of five trials in which a non-specific treatment protocol was used as a placebo found that the placebo performed as well as the specialized eating-disorder treatments, which included enhanced cognitive behavior therapy for eating disorders (CBT-E) and the Maudsley model for AN in adults (MANTRA) (Gutiérrez and Carrera, 2018). A meta-analysis of treatments for AN (Murray et al., 2018) concludes:

Current specialized treatments are more adept than comparator interventions at imparting change in weight-based AN symptoms at EOT [end of treatment], but not at follow-up. Specialized treatments confer no advantage over comparator interventions in terms of psychological symptoms (p. 1).

There is a problem, then, with the *status quo*. The paper just quoted argues that “A clear explication of core illness mechanisms, and their response to treatment, is a fundamental prerequisite for the development of precision treatments for AN,” and that “Elucidating the disconnect between weight and psychological outcomes in the treatment of AN speaks directly to this mission” (Murray et al., 2018, p. 1). The second observation may give us indirect insight into why the clear explication called for in the first remains elusive. Murray and colleagues review improvements in “weight-based symptoms” (by which they actually mean bodyweight alone) and improvements in psychological symptoms, but they do not consider behavioral improvements, despite voicing the common view that the psychology drives the physiology via the disordered eating behavior:

Psychological symptoms are thought to be central maintaining mechanisms in AN psychopathology, and this cluster of cognitions (e.g., overvaluation of shape and weight, fear of weight gain) is hypothesized to drive the behavioral features of AN (e.g., dietary restriction), which in turn drive weight loss (2018, p. 7).

Instead of drawing the obvious conclusion from their findings – that more specialized targeting of psychological symptoms yields no benefits – they instead conclude that even more focus on the psychology is needed:

Future treatment development efforts ought to adopt a specific focus on the more rapid relief of psychological AN pathology, such that the mechanisms by which they drive behavioral symptoms may be targeted and ameliorated throughout treatment (2018, p. 8).

Yet if the aim is really to “elucidate[e] the disconnect between weight and psychological outcome*s*” (Murray et al., 2018, p. 1), illuminating the role of eating behavior will be necessary to achieving it. Behaviors are a key mechanism by which body affects mind and vice versa. For instance, malnutrition directly increases the attentional salience of food-related stimuli in a physiology-to-psychology dopamine-mediated effect (for an overview, see Troscianko, 2017, p. 191-192). However, the cumulative effects of this heightened salience on everyday life are all mediated by changed behaviors: seeking out recipes, restaurant reviews, and food-related materials as replacements for or enhancements of actual eating; spending a long time shopping for food; cooking for other people without eating; and so on. These behaviors have powerful twofold effects: intensifying the psychological salience distortion while also exacerbating the physiological aspects of malnutrition. These effects may in turn have further effects such as the generation of cognitive dissonance, whose reduction may often involve *post hoc* alignments of cognitions with behaviors: for example, discounting the negative effects of secretive and restrictive eating for mind and body to justify continuing to eat in a disordered way. Thus, the vicious circle tightens.

In line with this pivotal role for behavior within the pathology of eating disorders, behavior-focused treatments seem to be as effective as those with extensive cognitive elements (Waller and Raykos, 2019); they are also much simpler and cheaper to provide, meaning that more patients have the chance to benefit from them. Accordingly, the case has recently been made from within the community of CBT researchers and practitioners (Waller and Raykos, 2019) and from outside it (Södersten et al., 2017) for a re-orientation toward treating the disordered eating behaviors. In this paper, we present the hypothesis that effectively treating eating is the key to treating eating disorders effectively: that behavior must be restored to the center of eating disorder treatment and research. We also propose, however, that the most meaningful model for both understanding and treating eating disorders is a dynamical systems model in which the interconnections between mind, body, and behavior are understood as structurally integral to the whole. Put simply: the eating has to be treated, but what shapes and is shaped by the eating cannot be ignored.

### A Critical Evaluation of the Psychological View

The psychology/cognition-first perspective has not emerged groundlessly, of course. It is supported by, and has in turn generated, a large amount of research aimed at uncovering correlations amongst eating disorders and other psychological disorders and traits, such as maladaptive perfectionism (Dahlenburg et al., 2019), borderline personality disorder (Miller et al., 2019), or mood disorders, anxiety disorders, alcohol and drug use disorders, and personality disorders (Udo and Grilo, 2019). Such correlations have indeed been found. Along with self-starvation, individuals with AN, for instance, may show florid psychological symptoms: depression (Voderholzer et al., 2019), anxiety (Kaye et al., 2004), obsessions and compulsions (Cederlöf et al., 2015; Levinson et al., 2019), delusions (Steinglass et al., 2007), psychosis (Seeman, 2014), attentional issues/hyperactivity (Yao et al., 2019), and autism (Sedgewick et al., 2019). Patients with eating disorders have also been found to have problematic personality traits such as perfectionism, neuroticism, obsessionality, negative emotionality, harm avoidance, low self-directedness, low cooperativeness, avoidant personality disorder, high constraint, high persistence, low novelty-seeking, high impulsivity, sensation-seeking, and borderline personality disorder (Vitousek and Manke, 1994; Cassin and von Ranson, 2005). The disordered eating behavior seems like one more comorbid symptom of a complex psychological disorder.

Of course, psychological factors matter. It is trivial to acknowledge that behavioral disturbances like eating problems may sometimes arise in response to complex or stressful situations which also generate psychological disturbances. It is equally obviously true that an individual will not engage in treatment at all without making a decision to pursue recovery, and that this decision-making is in part a psychological act. Given the ambivalence with which most people with eating disorders view both their illness and the possibility and desirability of recovery (Williams and Reid, 2010), this decision must not be taken for granted, either prior to the start of behavior change in treatment or at any point in the ongoing decision-making required to sustain change through treatment and in post-treatment life. But acknowledging the significance of these psychological factors is quite different from claiming that the “real” and “underlying” problem is a psychological one, for example at the level of enduring traits, and that treatment must proceed accordingly. As described above, the stronger claim has not yielded effective psychologically based treatments. In the rest of this section, therefore, we investigate five specific forms of reasoning that underpin it, and what makes these approaches both attractive and problematic.

#### Correlation Versus Causation

One reason why the psychological story persists despite its failure to generate effective treatments may be a confusion of correlation and causation. As noted, a multitude of correlations have been observed between eating disorders and other psychological problems. Once a correlation has been observed, a sensible next step is often to investigate possible *causal* links. Anxiety in general, and obsessive-compulsive disorder (OCD) as a specific expression of anxiety, are amongst the most common focal points for research on the causes of eating disorders (Matsunaga et al., 1999; Levinson et al., 2019). When national medical registry data were considered, however, a surprising finding emerged: an initial OCD diagnosis was indeed found to make a subsequent AN diagnosis more likely, but there was a 300% greater risk of an AN diagnosis that was followed by a subsequent diagnosis of OCD (Cederlöf et al., 2015). That is, the temporal relationship is the wrong way around for the AN to be an effect of the OCD. Similarly, when considering social anxiety disorder (SAD), some retrospective studies suggest that SAD often starts before an eating disorder (e.g., Bulik et al., 1997; Godart et al., 2003; Swinbourne and Touyz, 2007), but neither of the prospective studies conducted on the question (Buckner et al., 2010; Levinson and Rodebaugh, 2016) found a predictive role for SAD. Indeed, Buckner et al. (2010) found that a diagnosis of BN increased risk of a subsequent SAD diagnosis, not vice versa. In simple terms, too, the general idea that anxiety causes eating disorders needs to account for the far greater incidence rates of anxiety compared to the incidence of eating disorders.

The correlation/causation confusion is beginning to be acknowledged and tackled by recent observational studies which, instead of trying to identify diagnostic comorbidities between groups, take a dimensional approach that investigates relations amongst symptoms. Levinson et al. (2018), for example, studying a cohort with AN, found that “concern over mistakes was the only [personality or psychological] factor to have significant associations with the core dimensions of both AN and OCD” (p. 9). Similarly, investigating the relationships between eating disorders and OCD, Wu (2008) found that once a confounding general distress variable had been removed, apparent correlations between three OCD scales and eating disorders disappeared for AN, and only compulsive washing remained a correlate with BN, though a less powerful one than separate measures of panic and depression. Adding in a temporal element, in a 6-month prospective model Levinson and Rodebaugh (2016) found no prospective predictive power of SAD for the development of eating disorders, but they did identify maladaptive perfectionism as a shared prospective vulnerability for symptoms of both eating disorders and SAD. Social appearance anxiety also prospectively predicted eating disorder symptoms (here measured on three scales: drive for thinness, bulimia, and body dissatisfaction) but not did not predict social anxiety symptoms more generally. There are certainly some potential causal relationships worth investigating here, but the story emerging is nothing like the simple one in which OCD/SAD is the underlying cause of eating disorders.

These methods and findings show that asking more specific questions about symptom-level co-occurrence and time-course than the standard comorbidity questions yields more specific answers that change the terms of the debate: from asking which “disorder” precedes or causes which other “disorder,” to an approach centered on the search for lower-level patterns less freighted by the history of psychiatric categorization (see also Schaumberg et al., 2019). This kind of reorientation does not rule out the potential value of well-targeted psychological interventions: the causal relations between psychological and behavioral variables can and do flow both ways. Levinson and Rodebaugh’s (2016) results, for example, suggest that perfectionism could be a target for an intervention for both SAD and eating disorders. But we will argue that such interventions must always be attuned to the elucidation and resolution of current behavioral dynamics rather than being oriented around etiological history.

As we have shown (and see also Södersten et al., 2019b), then, the claim that eating disorders are mere surface manifestations of other underlying psychological problems is severely weakened by studies that offer more detailed time-course data and/or work with more granular conceptions of comorbidity. The conclusions generated by these increasingly sensitive observational data are also supported by experimental findings in which causation can be directly inferred. For example, “dietary restraint” is a concept developed to capture a concern with bodyweight control involving the intention to control food intake. High dietary restraint is an unstable state in which disinhibition and loss of control over eating is a likely result of changes in external food-related cues (Herman and Mack, 1975). Dietary restraint has been found to be directly modifiable by eating behavior, specifically eating rate (Zandian et al., 2009; see also Ioakimidis et al., 2009; Södersten et al., 2017). Similarly, the chronic anxiety associated with starvation in AN is reversible with appropriate short-term (Heruc et al., 2018) or longer-term (Bergh et al., 2002, 2013a) normalization of eating behavior. These findings suggest that at least some psychological problems are not causes of eating disorders, nor even comorbid correlations with eating disorders, but are actually *caused by* eating disorders. The idea that starvation triggers psychological symptoms is supported by a detailed experimental study of starvation and refeeding conducted during World War II, the so-called Minnesota starvation study (Keys et al., 1950). Here, young men selected for their robust good health were subjected to systematic semi-starvation, which induced in them severe psychological problems strikingly similar to those displayed by people with AN. Moreover, systematically refeeding the starving volunteers for 12 weeks, and allowing them subsequently to eat *ad libitum*, eliminated these problems within a year. There are certainly differences between the men in this study and the mostly women in today’s eating disorder trials. The individuals in the starvation study volunteered from a starting point of health and their rations were limited by the investigators, resulting in a managed and short-term though severe malnutrition, whereas the AN and BN patients have limited their own food intake, often extremely, sporadically, and/or protractedly, in the months or years before admission. However, in both cases, semi-starvation results in similar emotional and cognitive abnormalities that, along with the physiological damage, are reversed when eating behavior is fully normalized. The Minnesota study remains a critical piece of evidence in the field despite its age because arguably few more recent clinical studies have encouraged patients to regain enough bodyweight to make full physical, behavioral, and psychological recovery viable (Troscianko, 2014, 2016). We return to this issue and associated problems in the section “Feedback and CBT for Eating Disorders: Theoretical and Practical Problems” below. For now, it is interesting to observe that the search for the origins of eating disorders has so often turned so naturally to the psychological, and that the idea of physical/behavioral-to-cognitive causation may, despite substantial bodies of supporting evidence, appear so counterintuitive to many.

#### Other Factors

What other reasons, beyond the correlation/causation confusion, account for the persistence of psychology-oriented approaches to eating disorder treatment? We suggest four additional possibilities, the first following on directly from the correlation/causation question:

(1) A confusion of temporal starting points with focal points for treatment.

If one begins with the belief that “anorexia nervosa is the physical expression allowing the non-verbal expression of a generally important psychological disorder” (Lacoste, 2017, p. 80), as many researchers and clinicians seem to, it makes sense to try to get to the psychological “root cause” of all the symptoms. Most psychologically oriented therapies take roughly this approach. For example, in a randomized controlled trial comparing psychoanalytic psychotherapy with CBT for BN (Poulsen et al., 2014), the authors give a typical account of the rationale and process for any psychologically focused treatment. First the behavioral “symptoms” are attributed to a psychological “root”: “The treatment is based on the assumption that bulimic symptoms are rooted in a need to ward off inner feeling states and desires and in difficulties acknowledging and regulating such inner states” (2014, p. 110). Then the “symptoms” are relegated relative to the assumed “root,” which becomes the focus of therapy: the treatment is characterized by “involvement of the patient in a mutual reflection on the function of and the circumstances triggering the symptoms of the disorder. The bulimic symptoms are not necessarily discussed in every session, but the therapist assists the patient in understanding possible connections between the way that he or she eats and his or her affective state” (p. 110). More generally, the focus in many psychological treatments is on “helping patients understand the *meaning* of the manifest symptoms” (our emphasis), in the belief that the eating disorder is simply an expression of psychiatric developmental issues (Zerbe, 2001). This kind of therapeutic orientation may involve long-term (often years-long or even life-long) psychiatric excavation of past interpersonal trauma, sometimes in total absence of structured support for either changing eating behavior or weight restoration (e.g., Kadish, 2016), with the idea that discovering and tackling the ultimate mental origin will result in automatic resolution of its more “superficial” manifestations. As we have seen, however, this hope is not borne out by the evidence. In general, we can observe slippage between two meanings of *primary*: as an etiological starting point for a disorder, and as its most significant component, which deserves to be the focus of treatment. Identifying a plausible ultimate origin or proximate trigger, for example through first-person testimony, however, is not the same as identifying a neurobehavioral control mechanism for eating disorders.

Ignorance as to the ultimate causes of eating disorders is sometimes cited in explanation of our ignorance about treatments that work: “since it is unclear what leads people to develop eating disorders, it is consequently unclear how eating disorders should be treated (Giordano, 2002, p. 3). This might seem to make obvious sense, but we argue that it can in practice divert attention onto the issueless hunt for “root causes” at the expense of a pragmatic identification and elimination of dynamical maintaining factors.

(2) A tendency toward extremist, partisan thinking.

In many contexts of human endeavor, all-or-nothing thinking seems more appealing than careful exploration of the middle ground. Strober and Johnson (2012) criticize the intensity with which “entrenched viewpoints” are defended in eating disorder research and treatment, and argue that openness is needed to the multiple factors involved in the development of and recovery from an eating disorder. In practice, by contrast, debates about eating disorder treatment methods often involve statements to the effect that because one extreme of treatment is ineffective, the solution must be sought at the other extreme. For example, Lacoste (2017) states that “The psychiatric protocols with an excessive focus on weight gain are, for us, incomplete and ineffective” (2017, p. 80). He concludes that nutritional rehabilitation should be ignored in its entirety, and that instead, “research of the one or the several causes must be the major goal of an efficient psychotherapy” (p. 80). At the other end of the scale, the inhumanity of enforced refeeding protocols in some inpatient settings is an easy extreme to reject, especially since (as we discuss below) they are ineffective. Yet as we have begun to suggest, the zero-sum physical versus psychological stalemate may be broken by a conception of behavior as mediating between the two extremes.

(3) An intellectual preference for psychological over behavioral and physical matters.

“Psychiatrists listen to stories more than anything else they do” (Lewis, 2011, p. vii). The intellectual and emotional appeal of interpretive activity aimed at the human mind and human experience is probably a factor in many clinicians’ motivation to do what they do, not least in the field of eating disorders: “Behind every symptom of disordered eating lies a story that is longing to be told” (Zerbe, 2001). This applies as much in research as in treatment. Many research papers, particularly those on AN, are concerned ultimately with the *meaning* of the disorder (e.g., Giordano, 2002), and since meanings are conceptual, they are most easily located in the psychological realm. One common expression of intellectual appeal as motivator is the tendency to approach AN in particular as a mystery to be solved: researchers mention the “subtleties, contradictions, and paradoxes” of AN (Lask and Frampton, 2009), for instance, and often work from the starting point that it is a “puzzling and disturbing syndrome” (Chassler, 1994, p. 412). Whether in the service of research or treatment, storytelling about a difficult childhood or disentangling phenomenological anomalies offers easier footholds for complex interpretive activity than discussion of whether breakfast time should be moved forward by an hour. Psychological concerns may often be perceived as simply more interesting than physical or behavioral matters; it may disappoint the therapist that what really needs to be done this week is helping someone to include more fat in their diet. For the patient, too, psychologically focused “treatment” has the dual short-term advantage of being interesting and avoiding the anxiety-inducing activity of behavioral change.

(4) Clinical accommodations of anxiety in both patients and clinicians.

Intellectual appeal may be one side of the coin in the therapeutic encounter; anxiety is probably the other. Waller and Raykos (2019) observe that “Despite their being key to CBT-ED and other therapies, clinicians do not always use behavioral methods and are less likely to do so in response to their own anxiety levels” (2019, p. 189). Specifically, clinicians’ anxiety levels are inversely correlated with the probability of their using structured eating, behavioral experiments and other exposure techniques, and regular weighing. Patients’ anxieties (e.g., about weight gain) and associated safety behaviors (e.g., about changing eating habits) interact with clinicians’ anxieties (e.g., about distressing the patient), and the result can be an accommodation in which the clinician avoids pushing for behavioral change (Waller, 2011).

The likelihood of an ineffectually accommodating dynamic arising is increased by presence of mistaken beliefs about the power of the therapeutic alliance (Mulkens et al., 2018): if one believes that having a good interpersonal relationship with one’s patient matters, and also that making the patient do things (s)he does not want to do will jeopardize that, one is more likely to stay in the comfortable realm of talking about change. The first of these beliefs is supported by various reviews suggesting that therapeutic alliance can contribute to the efficacy of treatment for eating disorders (Antoniou and Cooper, 2013; Puls et al., 2019). However, the second belief is not substantiated. A meta-analysis conducted by Graves et al. (2017) suggests that in reality, symptom change and the therapeutic alliance are reciprocally related, with a stronger link between symptom change and subsequent alliance strength than vice versa. This implies that whatever is needed to bring about symptom change must be prioritized if the therapeutic alliance and its feedback benefits on further symptom change are to be fully exploited. Dropout is also a key mediating factor here (Graves et al., 2017), and is likely to be closely linked to successful symptom change or its absence. Overall, then, there is no reason to treat the therapeutic alliance and a behavioral focus as at odds with one another. What matters is that short-term anxieties be allayed by sustainable improvements in a patient’s health.

Other factors are doubtless at play, but these may be some of the primary contributors to how the behavioral gets sidelined in favor of the psychological in the treatment of eating disorders. In the following section, we propose a theoretical and practical model that offers a direct response and alternative to all these reasons for adherence to psychology-focused treatment protocols: the feedback model of pathology.

## A Feedback Model of Eating Disorders: Mind, Body, and Behavior

### Feedback and Mental Health

Feedback influences the dynamics of almost every natural and artificial process, from homeostatic temperature regulation to the flow of traffic through the internet. When the feedback is “negative” with a magnitude in the correct range, the system self-stabilizes: think of a cruise control system where current velocity is measured and compared with the reference velocity, and a signal sent to the engine to increase or decrease its output, resulting in a new velocity to measure – with shrinking fluctuations to reach 70 mph, and quick adjustments if the car starts traveling downhill. When the feedback is “positive,” by contrast, the system tends to become increasingly unstable, with small effects causing large changes: think of the microphone whose signal is amplified by a speaker too close to it, which feeds into the microphone which feeds it back into the speaker – resulting in a deafening screech. This problem persists until negative feedback intervenes, or the feedback loop is interrupted by an external force.

Feedback systems are best modeled by dynamical systems theory, which describes how any property of a system evolves over time. Any “real-world” system must be causal, i.e., future trajectories (mappings of how system properties evolve over time) must depend only on past states. Such systems may be deterministic, in which case, if the initial conditions are specified, the full trajectory can be uniquely determined. In most real-world cases, disturbances that cannot be predicted act upon the system. In this case the system is said to be stochastic, and only the average trajectory can be determined, since each specific trajectory is subject to an unpredictable set of disturbances. In general, however, one is interested not in a specific trajectory, but in all trajectories initiated within a set of initial conditions. Even more important than any specific trajectory, though, is long-term behavior: Systems may be stable (trajectories tend to a fixed point), periodic (trajectories oscillate infinitely, neither dying out nor blowing up), or unstable (trajectories continue to deviate from the initial condition at an increasing rate). Identifying long-term patterns of this kind allows for powerful predictions about, and interventions into, the dynamics of any system, including a human illness.

Eating disorders have long been understood as unstable feedback systems (Zeeman, 1976; Minuchin et al., 1978; Fairburn, 2008, p. 18-21; Kirsh, 2010; Cohen and Blaszczynski, 2015): as systems in which each effect has further effects, these further effects in turn cascading into further cause/effect cycles, all increasing the instability (as in the “vicious circle” of disordered behaviors, physical states, and cognitions we described earlier). Treatment is successful when it characterizes the feedback dynamics accurately enough to intervene in a way that disrupts them by introducing a source of stabilizing negative feedback.

This perspective automatically counters the tendency toward extremist all-or-nothing thinking [section “Other Factors,” point (2) above]. It is possible to apply feedback principles at any level of specificity: in eating disorders, for instance, one might focus solely on hormonal imbalance. However, the structural principles of dynamical systems encourage awareness of the multiple levels at which system dynamics interact, for instance of the fact that the hormonal system is coupled with the reward and stress systems, the wider metabolic system, etc. Equally, important feedback loops within the cognitive-emotional system may be identified, but it will rapidly become clear, when investigating them from a feedback perspective, that making sense of those dynamics requires insight into other factors driving and being driven by the cognitive-emotional patterns. Thus, a recognition naturally arises that cognition and emotion cannot be understood unless behavior and physiology are also understood: that “mind” is in constant feedback interaction with “body” and “eating behavior.”

In a systems-dynamic approach, the search for the original cause [see point (1) and the correlation/causation discussion above] is also misguided as a clinical endeavor, since temporal priority does not entail ontological or therapeutic priority. The quest for ultimate causal origins becomes insignificant relative to the question of how the current feedback dynamics are maintaining the problem^2^. The decision about where to intervene in a pathological feedback system is determined by pragmatic considerations focused on the present and the future, not the past. Such considerations include: (1) which factor is demonstrably maintaining most other factors and (2) which factor is most practically feasible to alter given the current circumstances. In AN, malnutrition tends to fulfill both criteria, since (1) it brings with it a vast cascade of cognitive, physical, and behavioral problems that create further cascading causal instabilities for the system, and (2) it is remedied with simple (if not easy) changes to eating habits. The same goes for restrict/binge/purge behaviors in BN. It is of course possible that some specific psychological component may satisfy both criteria, but if the question is asked and answered honestly, psychological therapeutic work in a malnourished state is rarely a feasible option, while behavioral work, such as modifying eating behavior, usually is. In fact, the American Psychiatric Association guidelines state specifically that “Attempts to conduct formal psychotherapy may be ineffective with starving patients” (Yager et al., 2006, p. 45). Therapy addressing the behaviors that maintain the starvation is far more likely to be effective.

A feedback model also addresses the problem of intellectual appeal [point (3) above]: once the pivotal role of behavior is understood in its theoretical context, it stops being boring. Sensitive modeling of pathological dynamics, including psychological states, allows the dynamics to be made predictable and hence tractable. This shift provides intellectual satisfaction for both clinician and patient. This perspective also at least begins to change the dynamics of anxiety and safety behavior within the clinical encounter [point (4)], both by making them explicit, precisely as a conspicuous and significant example of feedback dynamics in action, and by making unavoidably clear the impossibility of meaningfully disrupting the disorder while ignoring the eating behaviors.

### Feedback and CBT for Eating Disorders: Theoretical and Practical Problems

CBT is the best-known therapeutic instantiation of feedback principles. CBT manuals and theoretical discussions often include feedback-loop diagrams and the language of feedback principles (“processes” and “pathways,” “currently operating maintaining mechanisms,” “locking patients into a self-perpetuating state”; Fairburn, 2008, p. 18–20). For some individuals, CBT provides a helpful contribution to a recovery effort from an eating disorder. But if the feedback model is so powerful, and CBT is a direct formalization of it into therapeutic protocols, why are global remission and recovery rates so unimpressive?

The first problem comes back to the question of priority amongst the components at play in the feedback system. We discussed in the introduction, and can now reconsider through a feedback lens, the tension between cognitive and behavioral emphases. Continuing to use Fairburn (2008) as our case study, we find several feedback diagrams exemplifying the theory that guides the practice. In these diagrams (p. 19, 21), “over-valuation of shape and weight and their control” are presented as one component in a feedback loop in which all other components are structurally identical. In the accompanying text, however, the “core psychopathology” is given ontological priority. In dynamical-systems terms, the “overvaluation of shape and weight and their control” has been designated the *controller* of the system. In investigation of natural systems, it is common to speculate that one component is the controller and another is the thing being regulated. This is reverse engineering: given the full existing system, what are the components and how, when integrated, do they produce the overall behavior one has observed? But to move from speculation to demonstration requires extensive testing of the system dynamics to assess the relative strengths of multiple feedforward and feedback channels, eliminate confounds, etc. This type of research is highly compatible with more standard clinical testing. Indeed, the meta-analyses on treatment efficacy cited earlier suggest that to the extent that we have made progress in identifying the controller of the eating disorder system, we can be fairly confident that it is generally *not* the cognitions: the evidence we have suggests that creating ever more specialized psychological treatments will be ineffective. When asking why this evidence is often not acted on, it is worth noting another ambiguity in presentations of CBT methods and theory. In Fairburn’s (2008) discussion, etiological priority appears to be given first to dieting behaviors (“Anorexia nervosa typically starts in mid-teenage years with the onset of dietary restriction. […] Bulimia nervosa […] usually starts in much the same way,” p. 16–17) and then to aberrant cognitions (“central to the maintenance of the disorder is these patients’ core psychopathology: their dysfunctional schema for self-evaluation. […] [M]ost other features seen in the eating disorder can be understood as stemming directly from it,” p. 17). But there is slippage of the kind we described earlier [section “Other Factors,” point (1)] between identifying a past starting point and a current controller: phrases like “stemming from” work for both. It is easy to conflate the two, and the conflation is also manifest in the repeated use of the ambiguous term *secondary*, which can refer to both chronology and importance. This lack of clarity may in turn generate the impression that a search for origins can stand in for a search for the appropriate target for treatment.

These unresolved internal tensions at the heart of CBT for eating disorders can be added to the list of reasons we offered earlier for the wider clinical privileging of cognitive over behavioral treatments. The inconsistencies help explain why CBT has so far not fully capitalized on its dynamical-systems inheritance. Relatedly, they may be another contributor to the phenomenon of “therapist drift” in CBT for eating disorders (Waller and Turner, 2016). The “avoidance of evidence-based treatments” manifests above all in the failure to initiate behavior-focused aspects of treatment such as use of food diaries, encouragement of structured eating, and bodyweight monitoring (Mulkens et al., 2018). Perhaps this decision is not surprising if the cognitive overvaluation is presented as ontologically and clinically, if not etiologically, primary for the disorder.

CBT is working poorly, we suggest, in part because its tenets are poised uneasily between its cognitive and behavioral foundations, without a full espousal of the feedback principles that could resolve the tension. It is also the case that many clinicians do not provide true CBT and focus on the cognitive issues at the expense of the behavioral issues. Waller and Turner (2016) offer suggestions for countering this second problem at the individual level and at higher levels of practice in the field. But theoretical inconsistency and therapist drift are not the only factors involved in the relatively low efficacy of CBT for eating disorders. Many implementations of it (and of other non-CBT treatments) fall into other avoidable traps that systematically reduce its efficacy. We suggest that the most common additional problems are as follows:

(1) *Privileging bodyweight normalization over normalization of eating behaviors.* In restrictive eating disorders where patients are underweight, therapists may insist on weight gain at all costs, including strategies for intensive nutritional rehabilitation such as nasogastric tube feeding that involves no behavioral learning and may actually impede such learning. A review of the efficacy of tube feeding in AN suggests that short-term weight gain is generally achieved without concurrent improvement in psychiatric symptoms or evidence of long-term sustainability (Kells and Kelly-Weeder, 2016). A comparison of feeding methods in anorexia found no advantage in efficacy for tube feeding over ordinary oral intake of food or liquid supplements, and a wide range of disadvantages (Hart et al., 2013). In fact, Matusek and O’Dougherty Wright (2010) argue that the coercive nature of most tube feeding and associated tactics for enforced feeding loses its ethical justification if it does nothing to improve relapse rates. Meanwhile, in eating disorders where some patients are overweight, for example BED, recommended procedures might include energy restriction based on calorie-counting, even though such practices are well known to promote disordered eating (Simpson and Mazzeo, 2017).
(2) *Treating short-term bodyweight normalization as the primary measure of a successful recovery.* This emphasis may involve employing cursory and/or ill-defined criteria for “remission” and “recovery” that often rely heavily on BMI, rather than attending to a broader notion of recovery that includes normalization of eating behavior and daily activities of living. The urgent need for meaningful and standardized definitions of remission and recovery has been acknowledged (Khalsa et al., 2017; Tomba et al., 2019) but not widely acted on. Total absence of explicit remission/recovery criteria (e.g., McIntosh et al., 2005) is not uncommon, and wide discrepancies may also exist between clinical aims and the aspirations and values of patients (Darcy et al., 2010). Overemphasis on short-term bodyweight normalization, especially in AN, may also entail using inadequate follow-up protocols for tracking remission and recovery rates beyond end of treatment: follow-up beyond a year or two is rare. Short follow-up protocols have often been flagged as a problem, as they prevent accurate relapse estimates (e.g., Chakraborty and Basu, 2010; Amianto et al., 2015; Waller, 2016), but the practical and financial costs of continued monitoring of patients who have reached remission still often win out.
(3) *Underestimating the bodyweight values at which full recovery is possible.* Where remission and recovery are explicitly defined, and where BMI is used as a criterion, it is often a primary criterion. However, indefensibly low BMI thresholds are often set for end of treatment for AN, and avoidance of higher BMI values is often prioritized during treatment for BN, despite the fact that many patients with bulimia may present with suppressed bodyweight (Butryn et al., 2011). BMI levels of 20 or less are typical in AN treatment trials: a “healthy” or “recovered” bodyweight may be defined from as low as a BMI of 17.5 or 18.5 (Egger et al., 2016; Schmidt et al., 2016; Byrne et al., 2017; for an overview, see Troscianko, 2016). AN treatments often fail to sustain the weight gain of their patients, leaving them to be regarded as recovered when they have gained some weight, but only to around the BMI cutoff for “normal” or “healthy” bodyweight: AN may thus be diagnosed at similar BMI levels to those also being used to denote remission/recovery. The goal weight of AN treatment should be the weight at which physical and psychological symptoms are or can be resolved, but there is no agreed-upon BMI level at which this reliably occurs for all individuals (Lebow et al., 2018). This is partly thanks to the fact that BMI is a crude measure, which has some use as a rough proxy for physical restoration following malnutrition but is ill-suited to its common roles as an index of fatness (either overall body fat mass or its distribution) and as an index of health (Nuttall, 2015). In any case, an arbitrary and low standard for BMI recovery, based on the *low* point of a range widely considered “healthy” in a population-level normal distribution, is typically insufficient to suppress the risk of relapse.
  Finally, one additional reason for the inadequacy of standard weight/BMI targets in AN treatment is that they ignore evidence suggesting that temporary overshoot during weight restoration beyond an ultimately sustainable stable bodyweight level may be necessary for full and lasting recovery (Troscianko, 2014). The importance of temporary overshoot is suggested by a reanalysis of the Minnesota starvation study data, finding that fat mass is restored after malnutrition at a higher rate than fat-free mass, meaning that fat mass may require >100% restoration for fat-free mass to be fully restored (Dulloo, 1997; Dulloo et al., 1997). For most patients being treated for AN today, this outcome is impossible, because reduced “maintenance” diets are encouraged or imposed as soon as bodyweight reaches an arbitrary numerical threshold. In today’s climate, one can imagine the Minnesota volunteers never being permitted to recover fully from semi-starvation for fear that they might possibly get fat for a while. Here again, a mutual accommodation of patient and clinician anxieties may often be at play: “Physicians can be lulled into complacency by so-called ‘normal weights’ because we carry our own internal biases, including size and weight stigma. […] We must always resist our own learned, sizeist tendencies and continue to see the whole person in assessing their medical status” (Gaudiani, 2018, p. 90).

These interrelated problems may be caused in part by the institutional and funding pressures to which both clinical practice and academic research are subject. Whatever their causes, the adverse consequences for treatment success of a focus on normalizing BMI without altering eating behavior may be profound.

In theoretical terms, each of these three problems can be parsimoniously framed as deriving from an underestimation of the feedback interactions at work in eating disorders (devaluing the importance of normalizing all components of the mind–body–behavior system) as well as from misidentifying the controller of the feedback system (assuming it to be bodyweight). The second issue offers an interesting counterpoint to the general privileging of the cognitive components in eating disorders: here we see the physical issue (bodyweight) acquiring undue priority in significance and in treatment time-course. Almost nowhere in the treatment of eating disorders is eating *behavior* given priority – which, we argue, is the heart of the problem.

### A Behavioral Enhancement: Eating Rate

Of course, all aspects of eating behavior are not equally important. As we have seen, the implementation of cognitive-behavioral feedback principles in mainstream eating disorder treatments often emphasizes improvement in cognition or bodyweight at the expense of lasting behavioral change. But if a reorientation toward the improvement of abnormal eating behaviors were to happen, we would face the choice of which behavior it is most important to change first. One largely overlooked factor for whose relevance there is accumulating direct and indirect evidence across the eating disorder spectrum is *rate of eating*. Some of this evidence comes from obesity-oriented research. For example, rapid food intake is a strong risk factor for higher BMI (Leong et al., 2011; Ohkuma et al., 2015) and abdominal obesity (Nagahama et al., 2014), as well as predicting higher increases in bodyweight/BMI, bodyfat, and waist size in children (Ochiai et al., 2014) and adults (Tanihara et al., 2011). A slow eating rate has been shown to be associated with lower energy intake and higher perceived satiety (Andrade et al., 2008). Moreover, interventions to reduce eating rate have resulted in reduced energy intake and increased satiety amongst normal-weight participants (Shah et al., 2014). Slowing the rate of food intake also reduces energy intake without satiety change amongst those who previously had voluntarily eaten a large meal (Scisco et al., 2011). Mealtime feedback on eating speed helps obese children and teenagers to slow the rate at which they eat, reduce their energy intake while maintaining satiety levels, and allow them to lose bodyweight and fat and maintain that loss in the long term (Ford et al., 2010).

The mechanisms by which eating more slowly causes people to eat less food seem to be:

(1) The ability of changes in eating rate to change the secretion of the gut hormones PYY, ghrelin, and glucagon-like peptide that promote hunger and satiety (Kokkinos et al., 2010; Galhardo et al., 2012; Hawton et al., 2018).
(2) The increased receptivity of the reward areas of the brain to food stimuli when the eating rate is slowed (Hawton et al., 2018).

Thus, the intuitive notion that “gobbling” one’s food is associated with eating too much finds scientific support, and there is support too for the stronger causal claim that eating rate directly affects amount eaten, hunger and satiety signaling, and hence bodyweight regulation. As noted earlier, there is also evidence that eating rate directly affects psychological variables like dietary restraint: shifting a linear eating rate to a decelerated one reduced dietary restraint and broke the link between disinhibition and overeating amongst healthy women (Zandian et al., 2009). Thus eating rate seems a promising starting point for interventions to effect both psychological and physiological change.

### A Unifying Hypothesis, and a Successful Implementation

All these findings are relevant to the case of eating disorders, whether involving undereating (as in the restrictive subtype of AN), or overeating (as in BED), or a mixture (as in BN and the binge/purge subtype of AN). They suggest a simple hypothesis accounting for etiology, progression, and effective treatment across the full spectrum of eating problems, as follows.

Those who will develop the restrictive subtype of AN do not experience satiety during or after meals. Indeed, pro-anorexic online forums often include discussion of the strength of hunger cues and how to ignore them, some suggesting that high levels of hunger preceded the search for online “thinspiration.” In an attempt to control their bodyweight, they eat small amounts of food, and as a result their eating rate and hunger levels drop and their exercise levels increase, as the body’s survival priority switches from consumption to foraging (Bergh and Södersten, 1996; Södersten et al., 2008). Unlike most people, they are able to continue this behavior until they experience profound weight loss, deterioration of their physiological status, and deterioration of their psychiatric status. Many factors may contribute to this capacity, from a heightened sensitivity to the mood-enhancing effects of hunger to a greater concern with physical appearance as status marker: as ever, feedback dynamics mean that the effects of any such difference in initiating conditions may be rapidly amplified.

People who will develop BN or the binge/purge subtype of AN try to control their food intake but they cannot quite maintain their low level of consumption, possibly because reduced intake reduces eating rate and increases satiety and exercise levels less than in the restrictive eating case, or because they have higher levels of some subdimensions of impulsivity, like negative urgency (impulsive action intended to reduce negative emotions; Brockmeyer et al., 2014; Bardone-Cone et al., 2016) or sensation-seeking (Fischer et al., 2008). Those with BN eventually become very hungry, binge, and then purge. Those with BED replicate this strategy without purging. A further extension to the case of obesity may also be possible: individuals who will become obese eat too quickly to reliably experience satiety, so they continue to be hungry and continue to eat in excess of their metabolic needs (Ford et al., 2010; Galhardo et al., 2012).

All of these eating problems are driven by an inability to experience satiety due to rapid food consumption precluding the gut hormone changes that mediate satiety (Galhardo et al., 2012). Therefore, all eating-disorder patients can be effectively treated via a behavioral intervention that normalizes eating rate and thus satiety signaling. All additional aspects of the intervention should be tailored to support the normalization of eating behavior.

We acknowledge that our hypothesis may seem provocative. The question is, does the existing evidence support it? We believe that it does. As described earlier, global remission and recovery rates for CBT and other standard treatments are not good. An average of 24% of patients drop out of treatment (Linardon et al., 2018), roughly 37% of those who continue treatment reach remission (Hay et al., 2009; Hay, 2013), subsequent relapse rates are about 30% (Södersten et al., 2017), leading to true recovery for perhaps between 10% and 25% (Von Holle et al., 2008). These outcomes are subject to definitions of remission and recovery that are often lax and/or vague. In addition, they do not include individuals who were not treated because standard-care clinics often reject potential patients with AN if they have a very low BMI; their outcomes would not be reflected in these data.

Much better outcomes are seen with a treatment method focused on mealtime feedback. The Mandometer clinics originating at the Karolinska Institute in Stockholm, Sweden provide treatment in both inpatient and outpatient formats centered on a small device under a plate to measure food consumption and provide visual feedback to the eater on normal changes of eating rate and satiety over the course of the meal. In the case of eating rate, the eater’s speed is automatically compared with the desired normal eating rates for that meal, the two curves displayed to guide the eater in adjusting their rate of consumption. For satiety, the eater is prompted to input current satiety at intervals, and the resulting readings are likewise visualized relative to the normal satiety development for such a meal. The goal is for patients to learn to eat 300–350 g pf “ordinary Swedish food” in 10–15 min, by speeding up eating for those with restrictive eating disorders and by slowing it for those with overeating problems, and in both cases shifting it from a constant rate to a decelerated pattern. Regular use of the device is complemented by restricted exercise and warm rest after eating (in a warm room or with a thermal blanket).

The Mando clinics have an estimated 75% remission rate for over 1,400 patients treated for AN and BN after a median of 12.5 months of treatment, and 90% of those who reach remission go on to report full recovery (Bergh et al., 2002, 2013a; Södersten et al., 2019a). Furthermore, the standards for remission and recovery are detailed and ambitious. Remission requires: (1) normal eating behavior, including no binge/purge behaviors for 3 months in bulimia, and normal eating rate and feelings of satiety for all patients; (2) normal psychiatric status including symptoms of depression, anxiety, and obsession levels; (3) normal physiological profile; (4) normal bodyweight (BMI 19–24 for women, 20–25 for men), (5) no binge-eating; (6) stating that eating and bodyweight are no longer a problem; and (7) back to school or work (Bergh et al., 2013a). “Partial remission” is achieved when 5 of these criteria are met, and “recovery” requires maintenance of all seven criteria over the full follow-up phase, which includes 11 appointments over 5 years, each involving a 2 h semi-structured interview and consumption of a meal using the Mandometer without visual feedback (Bergh et al., 2002).

These high standards have been met by a strikingly simple implementation of feedback principles: normalizing the rate of eating and normalizing satiety regulation with mealtime feedback (Bergh et al., 2002, 2013a). As noted, in the case of AN, the rate of eating needs to be increased, and for BN eating rate needs to be slowed; in both cases a constant eating rate needs shifting to a decelerated pattern. In all eating disorders, awareness and responsiveness to the progressive transition from hunger to satiety during the course of a meal is also impaired (Halmi et al., 1989; Ramoz et al., 2015; Klastrup et al., 2019) and needs recalibrating alongside eating rate. All these changes can be achieved by means of the Mandometer device.

Importantly, after normalization of only eating behavior, without formal psychiatric therapy or drugs (all patients taking psychoactive drugs on admission are gradually withdrawn from them), all the psychological symptoms of AN and BN resolve. Unlike other attempts at treatment, these positive outcomes are long-lived, with only 10% relapsing over the 5-year follow-up. Moreover, the mortality rate during and after treatment has remained at 0%, despite the fact that many of the patients seen in this therapy are seriously affected individuals who have previously failed to reach remission at other clinics (Bergh et al., 2002, 2013a).

It is no accident that treating eating disorders as disorders of eating in this way should involve more robust criteria for remission and recovery than are typically employed. Some clinical researchers consider eating disorder patients to be in remission while they still express “residual” psychological, behavioral, and/or physical symptoms (Tomba et al., 2019). If the eating disorder is seen as a symptom of an underlying psychological problem, resolution of physiological and behavioral problems may be downplayed relative to the aim to uncover and address their “origins.” This treatment decision may mean that only partial recovery from the eating disorder itself is required: after all, if the eating problems are considered an effect rather than a cause of the psychological disturbances, their continuation in attenuated form may be considered relatively unimportant – perhaps on the view that a time lag is to be expected between resolution of the “causes” and clear-up of the “symptoms.” Conversely, if the focus of treatment is primarily on bodyweight normalization, continuation of both behavioral and psychological problems may be overlooked. Both approaches create ideal conditions for relapse (Tomba et al., 2019). If, on the other hand, the psychological problems are understood to arise as a direct consequence of disordered behaviors resulting in physiological destabilization, and are understood to contribute to sustaining that instability, then complete normalization of the physical, behavioral, and psychological issues must be required to signify remission, let alone full recovery. This therapeutic model makes it reasonable to require an absence of all symptoms associated with the dynamical system of the eating disorder.

Finally, it is worth noting the validation given within this definition of remission to direct self-report by patients: being able to say out loud to someone else, as part of the assessment of one’s recovery status, that “food and bodyweight are no longer a problem for me” involves being trusted, as a patient, to have meaningful insight into one’s own state of being, while also not giving that insight automatic primacy over all other measures. This treatment principle counters the easy assumption that a behavior-focused approach ignores personal experience: patients’ experiences and their ability to give testimony on them are here taken seriously within a squarely behavior-focused framework. There is no necessary conflict between a focus on behaviors and an appreciation of the reason why any of this matters: that individuals’ experiences of being alive can be enhanced. Indeed, the importance of *how* weight is normalized as well as that it is normalized is also testified to by patients who acknowledge that what they really need is to relearn how to eat. A statement to this effect was in fact the inspiration for the development of the Mandometer mealtime feedback method (Södersten et al., 2006).

### Expanding the Evidence Base

Despite the success of this approach to behavioral normalization, however, not all the factors on which the eating-rate hypothesis depends have been tested. For example, no prospective studies have been conducted to find out whether every eating disorder is preceded by rapid eating and lack of satiety, nor whether everyone who has anorexia eats too slowly or everyone who is obese eats too quickly (and all at a linear rate). We do not know what the most crucial differences are between people who keep eating less and exercising more (and so develop AN) and those who do not (and so develop obesity or another eating disorder), or between any of these cohorts and those who maintain healthy eating habits and bodyweight throughout their lives. It will be important to determine under what circumstances this idea holds.

We suggest that the most effective next step for testing our hypothesis would be to conduct fine-grained treatment research to establish the relative significance of eating rate normalization versus other aspects of the Mando treatment method, initially by separating out eating rate from satiety regulation training, exercise abstention, warm rest after meals, and verbal encouragements to behavioral change (see more on these latter in the next section). The first step in this testing could be to use a simple 2 × 2 design balancing eating rate (ER) against the four other covariates (4CoV), and so including four conditions:

1: 4CoV and ER.
2: not 4CoV and ER.
3: 4CoV not ER.
4: not 4CoV not ER.

Involving a clear prediction as to the ranking of efficacy by condition (in the order presented) and disambiguating between eating rate and the other components, this design would provide newly structured evidence on Mando efficacy, and would set the stage for testing each of the covariates in order to assess their inner structure. An external comparator (e.g., an appropriate treatment course of CBT, CBT-E, or other treatment-as-usual) could also be included. With appropriate longitudinal assessment of symptom change, a study of this kind would further the aspiration set out by Lampard and Sharbanee (2015) to identify the “active ingredients” of treatments rather than viewing treatments as irreducible units.

This research would have practical and theoretical benefits as regards the interrelations between Mando and CBT. It would ideally be conducted by teams including specialists in CBT as well as the Mando practitioners and researchers. At present all Mando evidence has been gathered by the Mando teams in their own clinics, and most research publications on the Mando method have been published by the developers of the method (with the exception of one problematic study, van Elburg et al., 2012; for a response, see Bergh et al., 2013b). This research would thus help expand the research on the Mando method in scope and disciplinary grounding. It would also shed light on the specifics of practitioner engagement with the patients, to address the question of precisely what overlap exists between the forms of interpersonal support offered in CBT and Mando. This in turn would broach the broader question of whether the model we propose here is best thought of as a new alternative to CBT or a return to the behavioral foundations of CBT. Both methods emphasize the significance of behavioral normalization in the treatment of eating disorders, and CBT involves some acknowledgment of the importance of feedback dynamics. But it is only through careful explorations of the details of how both methods operationalize these principles that we will arrive at a meaningful characterization of the similarities and differences between them, and an answer to the question of how exactly the present theory relates to the theory and the practice of CBT.

This interventional study focused on the treatment and recovery process could be complemented by a prospective observational study in which eating rate were tracked at regular time points against satiety regulation, dietary intake, exercise habits, bodyweight changes, and ED onset (where applicable), around the mid-adolescent phase where ED onset is most common, to assess the prospective predictive power specifically of eating rate in the etiology of eating problems. Studies of these kinds would contribute to modeling the feedback dynamics of health and illness and of successful and failed recoveries. As such, they would enrich the existing evidence base for the treatment of eating problems, which currently suggests that eating disorders and other problems with bodyweight and eating can be effectively treated using protocols that focus on the simple behavioral retraining of eating rate.

Those who treat AN, BN, or BED often regard these conditions as arising primarily from either a personality problem or a psychological problem. Treatment often proceeds on this basis, or else focuses on weight normalization at the cost of all other factors. However, there are good reasons for thinking that normalization of eating behaviors, rather than of bodyweight *per se*, is the key driver of recovery. After all, the “semi-starvation neurosis” seen in AN is also present, not only in obese people whose BMI is reduced, but in obese people with BED with no bodyweight loss, and also in people with BN, who often have a normal bodyweight (Södersten et al., 2008). The main driver of improvement in all of these problems may be the normalization of eating behavior, rather than nutritional rehabilitation (e.g., via nasogastric tube) irrespective of behavior. A common principle for all these eating problems is that the rate at which food is consumed is a strong contributor to regulating the amount of food that is eaten and the experience of eating it. Standard care could be enhanced by a changed behavioral focus in treatment, accompanied by improved standards for remission and recovery. This change in therapeutic emphasis would parallel the emerging consensus in treatment of addiction and substance abuse that although psychological therapy might be the individual’s preferred way of tackling (or avoiding tackling) the behavioral problems, effective treatment is founded on the fact that the abnormal behavior sustains the psychological difficulties, not *vice versa* (Carroll and Onken, 2005; O’Brien, 2008). Overall, we propose that a shift away from the extremes of physiology (bodyweight) or psychology toward eating behavior as their structural intermediary may resolve many of the problems manifest in eating disorder treatment today.

## Complicating the Simple Story

There is substantial evidence for the success of a behavior-focused treatment of eating disorders that normalizes both (1) eating rate and (2) experiences of mealtime satiety. This treatment protocol, as implemented over more than 20 years, has also involved two additional behavioral practices: (3) restricted exercise and (4) warm conditions in which to rest after eating. Both strategies help retain calories for weight gain in patients with AN, rather than losing them to heat production or exercise; warm rest after meals also helps counters post-prandial anxiety in all patients (Bergh et al., 2013a; Södersten et al., 2017). The four elements have not been separated out for individual efficacy tests (such as could be initiated via the protocol we set out in the previous section), and they have been implemented in concert only in specific clinics (although enforced rest is widespread in inpatient treatment for anorexia, and ambient warmth may be a feature of some). These clinics may be distinct from others in ways that are easily quantifiable or not. But the strikingly improved remission and recovery rates for the full spectrum of eating disorders suggest that the dual-track eating-speed-plus-satiety mealtime feedback, with or without warm rest and exercise restriction, is what makes the difference.

These findings could generate a strong claim that psychological therapy is not needed to successfully treat an eating disorder. If “psychopathology is considered a consequence, not a cause, of starvation” (Bergh et al., 2002, p. 9486), then one might argue that addressing the starvation is all that is needed. On this view, anything else likely to help would amount to common-sense encouragement of the crucial behavioral changes being made: “cognitive therapy can be considered to be good advice similar to that given to patients whose eating behavior is being normalized” (Södersten et al., 2017, p. 187). However, this conclusion may be going too far. The Mando team’s description of the process of “social reconstruction” from which patients benefit along with the core treatment sounds a lot like CBT: helping the patient to understand the mechanisms maintaining their disorder, to practice more acceptant thoughts and attitudes, regulate emotions, enhance interpersonal skills, and so forth. Much of this type of treatment, of course, is also common sense, but CBT, too, works not least by appealing to common sense. Encouraging a patient not to take every sensation or emotion at face value, but to interrogate its possible causes and effects and challenge it where appropriate, is a common-sense way of proceeding. Asking what the manifold reasons might be why I “don’t feel hungry” rather than assuming the reason is “I don’t need to eat”; asking what the consequences of eating and not eating because of a perceived lack of hunger might be; and resolving on a course of action as a result – all this is both common sense and CBT. Expertise in providing CBT consists not least in anticipating likely patterns of cause and effect, and in encouraging the appropriate conclusions to be not just drawn but acted on. These therapeutic goals are also common sense that has been formalized into therapeutic method. On a smaller scale, the “behavioral approximations” used to help patients start to eat (from food on plate to empty fork in mouth to smelling food on fork, etc., Bergh et al., 2002) are common sense, formalized in a different tradition: behaviorism. Whether one thinks they deserve the label “therapy” or just “common sense,” all such encouragements to change are, in the Mando treatment, designed to facilitate the normalization of eating behavior through simple visual feedback during eating. Just as with the four behavioral components of the Mando method, this fifth element will require in-depth descriptive and efficacy analysis to clarify the nature of its role in generating lasting behavior change as part of an effective mind–body–behavior intervention.

## Discussion: Putting It All Together

Minds are always embodied, and no solution to a problem involving embodied minds in action can ignore any part of the mind–body–behavior constellation. The question is how to balance their requirements effectively at different phases of treatment. The behavior pole has typically attracted the least clinical interest in both the study and treatment of eating disorders, possibly because it lacks (for clinician and/or patient) the appeal of measurability that a focus on the physiological factors allows, as well as lacking the appeal of intellectual fascination and the appeal of legitimized procrastination that focusing on the psychological factors satisfies. As we have seen, however, approaches that put specific eating behaviors center-stage while acknowledging realities that are also partly psychological, such as the need to relearn how to eat and feel hungry before eating and full after eating, show the most promise for success. And as Lampard and Sharbanee (2015) observe, the point is not to pit hermetically sealed treatment protocols against each other to see which wins; the goal is to find out what the active ingredients are in any therapeutic tradition, and to make sure we use more of them and less of the things that aren’t needed and that reduce patients’ belief in the possibility of recovery.

It may also be worthwhile looking further afield for ways to maximize therapeutic efficacy – and to increase uptake of and full engagement with therapeutic support. After all, as we noted earlier, any change in behavior must be preceded by a decision to change. Exploratory research suggests, for example, that reading habits can have significant effects on factors central to the development and maintenance of eating disorders, including mood, self-esteem, feelings about one’s body, and diet and exercise habits, in both helpful and harmful directions (Troscianko, 2018). The effects often manifest via contributions to (usually harmful) positive feedback loops or (usually beneficial) negative feedback dynamics, via a wide range of intermediary mechanisms. These include: increased or decreased obsessiveness; constructive or unconstructive comparisons with literary characters; helpful distraction from the difficulties of eating or exacerbation of the guilt of not exercising; inspiring prompts to explore embodiment and sexuality, or misleading distortions of bodily sensations; and even the phenomenon of deliberate self-triggering (seeking out books specifically to exacerbate one’s eating disorder), often relying on highly selective interpretation of textual material (Troscianko, 2017).

The wide-ranging effects of reading literature are just one example of the power of cultural phenomena to intervene at every point in the mind–body–behavior feedback systems of disordered eating. The things people read, watch, listen to, and are confronted with on their way to work or their time on Instagram all have the potential to make part of the difference between spiraling further into illness or understanding that the time has come to leave illness behind. Thus they can all be part of the motivation to seek out treatment in the first place, to persevere through the physical and mental discomfort of recovery, and to maintain and build on the resulting good health – or to espouse disordered habits and retreat back into seductive but damaging exercises of self-control or self-objectification.

**FIGURE 1:**
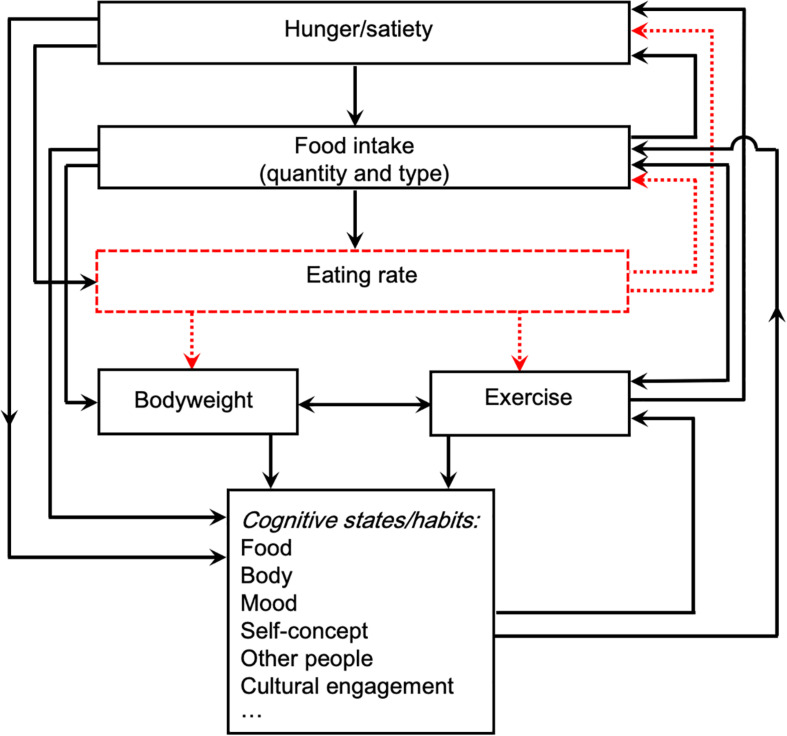
Feedback interactions amongst cognitive, physiological, and behavioral components of the dynamics involved in eating. The red dotted box indicates the controller of the system, here eating rate, which provides an effective means of intervention in the feedback system, e.g., via the Mandometer device. The red dotted lines indicate the primary causal effects of controlling eating rate. Specific intermediary mechanisms (e.g., hormonal mediators) are omitted here for clarity.

Crucially, it is wrong to see cultural, social, or aesthetic factors as widely divergent from physiological or behavioral ones. The bottom line is that no eating treatment works if people do not accept or adhere to it, and cultural, social, and aesthetic factors always play a role in determining this, just as physiological or behavioral factors do. Take food choice as an example. While eating rate may be a critically important mechanism for the control of eating, and overall energy intake is central to the development of malnutrition and its reversal, specific food choices also play a role in eating disorders and recovery. Causality is not always easily established, but lack of dietary fat, for instance, has been repeatedly associated with specific physical and cognitive deficits found in underweight or post-underweight individuals (Mayer et al., 2012; Nguyen et al., 2019; for an overview, see Troscianko, 2020). Ultra-processed high-sugar foods are implicated in binging behaviors (Ayton and Ibrahim, 2020), while diets higher in fat and protein may offer psychological and physical benefits post-recovery from a restrictive eating disorder (Troscianko, 2012). Higher-fat and -protein diets may also align neatly with the neural and hormonal mechanisms by which eating-rate interventions are effective for overweight, by modulating ghrelin and leptin levels (Lomenick et al., 2009; Ebbeling et al., 2018) and secretion of glucagon-like peptide (Gibbons et al., 2013). Yet the world is full of good reasons to eat sugar, from the social significance of a large family meal to the sensory and nostalgic pleasures of a chocolate Easter egg, and in an everyday sense as part of the wider importance of being relaxed and open about food during and after recovery from an eating disorder. The world is also full of discouragements to eat fat, via almost every “healthy-eating” site and food label, questionable as that advice is in physiological (Heileson, 2020) as well as psychological respects. Such advice, and its close associations with specific body ideals, may be hard to ignore for someone who has long exercised high levels of “dietary restraint” involving loss of sensitivity to physiological cues in favor of cognitive ones (including preoccupation with dietary advice and culturally validated beauty ideals). Thus food choices depend on, and in turn influence, multiple aspects of the dynamical eating disorder system, from social media habits to the experiences of hunger and satiety. No physiological or behavioral model can be meaningful if it ignores these complex interplays, of which we give a schematic outline in Figure 1.

Other examples of the need for thinking in terms of dynamical feedback systems could be enumerated. Framing physical movement as a “scenic walk” rather than as “exercise,” or perceiving a race as more fun, makes people less likely to eat as much dessert or choose an enjoyable snack afterward (Werle et al., 2015). Treating patients as individuals, with respect and empathy, and teaching them specific skills to support a return to the “real world” (Patterson et al., 2018), as well as communicating clearly about the goals of treatment (Sibeoni et al., 2017), may determine whether they drop out of treatment for AN or not, and whether they benefit from it lastingly or not. Apparently trivial practicalities can also be make-or-break factors: in the trial of an eating-rate intervention for obese children (Hamilton-Shield et al., 2014), low participant engagement came down to widespread technical issues. Of similar importance were more subjective failures of the device to engage young users, who complained about the voice commands being annoying, boring, robotic, and an American male adult voice; instead they wanted a cartoon voice, a range of voice options to choose from, the voice of another child, or their own voice. Turning the device into more of an interactive game could have helped keep children and teens engaged for long enough for the benefits to become self-sustaining. It is easy to neglect the sheer complexity of the interactions involved in eating.

Any treatment model that ignores the cultural and the social worlds will fail at least some of the time. So will any treatment model that ignores the individual’s context – in any respect from personality to religious background to age or length of illness. Eating disorders are sometimes defended as valuable manifestations of diversity or freedom of choice, despite the obvious forms of damage they involve – so the concepts of treatment and recovery are themselves controversial to some (Fox et al., 2005). But these apparently complex facts do not diminish the truth that the essence of a successful treatment for eating may be a resolute focus on simple, universal parameters of the eating itself. A simple human–machine system – a small device under a plate providing visual feedback to the eater – is a microcosm of the feedback loops that spiral across the entire human world.

This contribution to solving the problem of eating disorders is a long way from the divergent forms of apparent common sense encapsulated in the extremes of force-feeding or the unraveling of deep-rooted dysfunctions. We suggest that the alternative to these demonstrably inadequate perspectives is a view in which the right behaviors, pinpointed and healed, act as a powerful fulcrum between world, body, and mind.

## Data Availability Statement

The original contributions presented in the study are included in the article; further inquiries can be directed to the corresponding author.

## Author Contributions

ET and ML contributed to the development of the ideas presented in this manuscript, as well as to drafting and revising it. Both authors contributed to the article and approved the submitted version.

## Conflict of Interest

ML owns stock in the Mando Group. The remaining author declares that the research was conducted in the absence of any commercial or financial relationships that could be construed as a potential conflict of interest.

## Footnotes

1 Over the past couple of decades, *cognitive* has been gradually winning out over *psychological* (and *mental*) as the descriptor of choice for qualifying the mind and its activities, except in clinical contexts, where the adjectives *psychological* and *psychiatric* are frequently used to characterize disorders or symptoms. In this article we use the term *psychological* as a neutral descriptor for mind-related characteristics, and the term *cognitive* when discussing those aspects in the context of cognitive behavioral therapy. For the purposes of our discussion we consider the terms interchangeable. We assume both to include emotional processing and everything else the mind does.

2 From a prevention perspective, identification of initiating factors is obviously important, and one useful distinction here is between one-off inputs which do not have ongoing roles within a system, and inputs which become integrated into the feedback dynamics as components or controllers.
